# SG-APSIC1204: Time-based deisolation of generally asymptomatic immunocompetent COVID-19 patients on day 8 of infection to clean wards is safe

**DOI:** 10.1017/ash.2023.17

**Published:** 2023-03-16

**Authors:** Nazira Binte Muhammad Fauzi, Revathi Sridhar, Chan Hwang Ching, Annie Poh, Isaac Low, Dale Fisher, Jyoti Somani

**Affiliations:** National University Hospital, Singapore; Singapore National University Hospital, Singapore; Singapore National University Hospital, Singapore; Singapore National University Hospital, Singapore; Singapore National University Hospital, Singapore; Singapore National University Hospital, Singapore; Singapore National University Hospital, Singapore

## Abstract

**Objectives:** The National University Hospital (NUH) is a tertiary-care teaching hospital in Singapore with 60% of patients in 6–8-bed cubicles. NUH recently changed to a time-based deisolation criterion for immunocompetent COVID-19 patients in cohort wards who are afebrile and improved but did not meet the antigen rapid test negative criteria at day 5–6 and who required continued hospital care. The MOH guidelines and studies of viral load trajectory from the SARS-CoV-2 δ (delta) variant suggest that by day 8 of infection, viral loads drop and the risk of transmission is low. We defined a cycle threshold (Ct) value ≥25 as the point at which virus cultures are negative. We assessed whether a time-based deisolation at day 8 correlated with Ct ≥25 during the SARS-CoV-2 ο (omicron) variant pandemic surge. **Methods:** Data for patients and staff with confirmed positive COVID-19 PCR between January to March 2022 were collected. These data comprised a convenience sample collected retrospectively by the epidemiology team and the obstetrics and gynecology team and were used to deisolate patients. Nasopharyngeal (NP) swabs were sent for PCR for all admissions, to confirm diagnosis, for deisolation and/or transfer, and for staff suspected to have COVID-19 as part of hospital staff policy. **Results:** Overall, 403 observations were obtained. For 145 NP swabs tested by SARS-CoV-2 PCR on day 1, the median Ct value was 19.55 (IQR, 9.01). The median Ct for 87 observations on day 2 was 15.95 (IQR, 3.45). The median Ct value for 14 observations on day 8 was 24.22 (IQR, 5.19). From day 9 to day 37, with 47 observations, the Ct was generally >25. **Conclusions:** During the SARS-CoV-2 ο (omicron) surge, NP swabs sent on day 8 had a median Ct value of 24.22. After day 8, the median Ct was >25. The discontinuation of isolation precautions on day 8 balances the use of dedicated COVID-19 beds with risk mitigation of transmission for recovered patients who require ongoing hospitalization. Small sample size and heterogeneous reasons for testing NP swabs after day 5 likely skewed our results and limits the generalizability of our results.

